# Experiences of the implementation of a tool for lifestyle intervention in primary health care: a qualitative study among managers and professional groups

**DOI:** 10.1186/1472-6963-11-195

**Published:** 2011-08-18

**Authors:** Siw Carlfjord, Agneta Andersson, Malou Lindberg

**Affiliations:** 1 Department of Medical and Health Sciences, Division of Community Medicine, Linköping University, SE-581 83 Linköping, Sweden; 2 R&D Department of Local Health Care in Östergötland, Linköping University, SE-582 24 Linköping, Sweden

## Abstract

**Background:**

In recent years there has been increasing interest in transferring new knowledge into health care practices, a process often referred to as implementation. The various subcultures that exist among health care workers may be an obstacle in this process. The aim of this study was to explore how professional groups and managers experienced the implementation of a new tool for lifestyle intervention in primary health care (PHC). The computer-based tool was introduced with the intention of facilitating the delivery of preventive services.

**Methods:**

Focus group interviews with staff and individual interviews with managers at six PHC units in the southeast of Sweden were performed 9 months after the introduction of the new working tool. Staff interviews were conducted in groups according to profession, and were analysed using manifest content analysis. Experiences and opinions from the different staff groups and from managers were analysed.

**Results:**

Implementation preconditions, opinions about the lifestyle test, and opinions about usage were the main areas identified. In each of the groups, managers and professionals, factors related to the existing subcultures seemed to influence their experiences of the implementation. Managers were visionary, GPs were reluctant, nurses were open, and nurse assistants were indifferent.

**Conclusion:**

This study indicates that the existing subcultures in PHC influence how the implementation of an innovation is perceived by managers and the different professionals. In PHC, an organization with several subcultures and an established hierarchical structure, an implementation strategy aimed at all groups did not seem to result in a successful uptake of the new method.

## Background

In recent years there has been increasing interest in how to transfer new knowledge into health care practices, a process often referred to as implementation. Important factors shown to influence implementation processes are innovation characteristics, adopter characteristics, context, and implementation activities [1]. Implementation of an innovation is described by Rogers as an innovation-decision process with a knowledge stage, a persuasion stage, a decision stage, an implementation stage and, finally, a confirmation stage [2]. A number of barriers to implementation have been identified, factors that should be taken into account at the introduction of evidence-based practice (EBP) in health care [3]. Some of these barriers are related to the new practice at hand, the organizational context or the health care system; others relate to the individual practitioners and the health care team [3]. Factors related to the health care team or the practitioner can be individual, such as motivation, attitudes and knowledge, but could also include the fact that different health professions respond to different forms of evidence [4]. Interprofessional collaboration in teams is important to provide good quality health care [5]. However, throughout history, professions have struggled to define their identity and role in patient care, and each profession has their unique subculture, a kind of professional identity, including values, beliefs and behaviour [5,6]. The differences between subcultures in health care organizations seem to be well established [4-8], but knowledge about how these subcultures influence the implementation of new practices is limited. Most studies evaluating how professionals respond to efforts to implement new methods in health care focus on physicians. A systematic review of health professionals' perceptions of the implementation of shared decision-making in clinical practice conclude that little is known about any health professionals other than physicians [9]. An update of the review found no signs of a more interprofessional approach to the subject in recently published articles [10]. An Australian study of policy implementation found that different values among medical and nursing professions, reflecting different training, influenced the implementation process [11]. However, the scientific evidence is limited and there is a need to further explore if and how existing subcultures in health care influence implementation.

The study described in this paper was conducted in primary health care (PHC) in Sweden. PHC is a suitable arena for providing services that promote health and prevent illness, a task that has become more important because preventable diseases are increasing worldwide [12]. Due to lack of time, resources and skills, however, preventive services are not provided by PHC to a degree that corresponds to the needs of the population [13-15]. Today, modern technology can be used to facilitate health promotion in PHC, and computer-delivered interventions have been evaluated and found effective in various settings [16,17]. However, in order to be useful in PHC settings, tools like these have to be implemented and integrated into routine practice [18]. Recently, a computer-based tool for lifestyle intervention was introduced at a number of PHC units in Sweden, with the intention of facilitating the delivery of preventive services [19]. This provided an opportunity to study the implementation, and a qualitative study focusing on how different professions experienced the implementation was performed. The aim of the study was to explore how professional groups and managers experienced the implementation of a new tool for lifestyle intervention in PHC. This article reports how the implementation was perceived by the different professionals, and discusses how professional subcultures in health care influence implementation.

## Methods

The study was conducted using a qualitative descriptive design, including focus group interviews and individual interviews [20,21]. Six PHC units (i.e. health care centres with general practitioners (GPs) and other staff members) in three different county councils in Sweden participated in the study. A computer-based tool for lifestyle intervention was introduced at the units, and staff members were instructed to refer their patients to the computer. The lifestyle test included questions about alcohol consumption and physical activity, and patients who completed the test received their results and tailored advice from a printer connected to the computer. The computer-based test is described in detail by Carlfjord et al. [19]. During the study period all units received regular written feedback provided by the research team, showing the number of patients who had performed the test, the proportion of patients with risky behaviour and the proportion of patients reporting having been referred to the test by staff.

### Data collection

When the computer-based test had been in operation for 9 months, the manager at each unit was invited to an individual interview. All six managers participated in the individual interviews; five of these were held at the PHC units, and one, out of convenience, in a room at the university. Among the managers, three different professions were represented: one physician, four nurses and one physical therapist. Two of the six managers were men. Individual interviews with managers lasted between 18 and 28 min (average 22 min) and were conducted by S.C.

Staff members were invited to participate in focus group interviews after 9 months. All staff members who had direct contact with patients, and thus could be expected to have had the opportunity to refer patients to the computer (in total 159 individuals), were contacted. Those who signed up for the interviews and showed up at the interview session were included as informants, and considered a volunteer sampling. The different staff categories were interviewed separately. At each unit, GPs formed one group, nurses another group and allied professions (APs) formed a third group together with nurse assistants (NAs). APs were represented in only two of the groups, and included one dietician, one welfare officer, one occupational therapist and one psychologist. In total 16 focus group interviews and two individual staff interviews were conducted, involving 67 staff members (25 GPs, 27 nurses, 11 NAs, 4 APs). One of the nurses and 12 of the GPs were men; all other participants were women. Focus group size varied from two to eight members; the average was four. The staff interviews were conducted on the premises of each PHC unit in a room used for staff meetings. Focus group interviews lasted between 35 and 48 min (average 37 min); one author (S.C.) served as moderator and the interviews were observed by an assistant taking notes. After each session the moderator and the assistant had a brief talk about their impressions. This method of moderator/assistant roles is described by Krueger [21]. Individual staff interviews were performed when only one member of a certain staff group could participate (NAs at two of the units); these lasted 15 and 17 min. Interviews were performed between January and June 2009.

The interview guides covered the following topics: the overall working situation coinciding with the implementation process, experiences of the implementation activities and of the computer-based test, how to address lifestyle issues with patients and openness to innovations at the unit. Similar guides were used for staff and manager interviews.

Interviews were recorded using a digital recorder and were transcribed verbatim. S.C. transcribed four of the interviews and the others were transcribed by an assistant. After transcription, S.C. listened to the recordings and read all the transcribed material, and made corrections if necessary.

### Data analysis

Data were analysed using manifest qualitative content analysis, a method that describes visible, obvious content, but also allows interpretation of a text [22,23]. The narrative text was read and re-read to obtain an overall picture and to capture essential features. Meaning units, that is, words or sentences that are related to each other through content or context, were then identified throughout the text [22]. The meaning units were condensed to contain only a few central words, interpreted and labelled with suitable codes. Codes were then sorted into categories emerging from the interviews. This process was conducted by all the authors (S.C., A.A., M.L.) and was discussed until consensus was reached. The goal was to explore experiences, and statements expressing values, beliefs and behaviours were of particular interest in the analysis. Statements originating from each profession were treated separately, enabling experiences from the different groups to be compared. Due to the fact that only 4 APs participated in 2 groups together with NAs, AP results were not included in the analysis.

### Ethics

The study was approved by the Ethics Board in Linköping, Sweden (Dnr Ö 16-08). Respondents were informed that all data collected would be treated confidentially, and the results presented so that no individuals can be identified.

## Results

The results presented are representative of the staff groups but differing opinions are also presented. The results are supported by quotations from the interviews. Three main areas were identified: implementation preconditions, opinions about the lifestyle test, and opinions about usage.

Quotations are selective and illustrative. Words left out in a quotation are marked with [...], author comments to clarify quotations appear in brackets [ ], and ... means hesitation.

### Implementation preconditions

Three categories were found regarding implementation preconditions: expectations (before receiving the tool), involvement (in the decision to receive the tool), and the introduction (perceptions of how the new tool was introduced at the unit).

With regard to expectations, the managers were unanimously positive about the new tool, expressing openness and curiosity. They perceived prevention as an important task and felt a responsibility to provide preventive services. In general, they saw great openness to change and interest in innovations among their staff, but mentioned reluctance in certain groups, especially GPs, or specific individuals. Managers mentioned involvement in terms of a discussion with the staff group. Information was perceived as relevant, but probably would have been more effective if given to smaller groups. The manager group stated that it takes time before a new way of working becomes routine, and they found this reasonable. Managers also mentioned structural dimensions of the implementation, such as the lack of a local plan for action, having overseen the importance of a special person in charge at the unit, and the possibility of adding a certain degree of obligation to the implementation process.

If I'd had more time I would have tried to do more, so that we had set some goals for this - that we pushed the issue; that we were forced to refer a certain number per person, per month, ... so that there was some mild coercion.

Positive expectations among managers were not shared by the GPs, who gave a different view, expressing indifference and lack of enthusiasm, and pointing out that they have enough to do without the tool.

We were a bit sceptical [...] we have enough to do anyway and because we should probably take care of our own work first.

The perception of constantly having a heavy work load was common, GPs seemed genuinely tired of never-ending changes and many changes were perceived as threatening to their independence. They welcomed medical innovative changes for their own profession, but were reluctant about other innovations. GPs did not feel involved in the decision to receive the lifestyle computer, they seemed indifferent to the new tool, and saw no reason to welcome or to reject it. When GPs mentioned the information, they described it as good, but as being intended for someone else. They felt no need for the tool, as they already integrate lifestyle issues as part of their daily work, and they suggested that specific information should be given to those concerned. GPs acknowledged the importance of lifestyle issues, and indicated that nurses and physical therapists have key functions in this work.

It feels a bit better to be able to refer them to our lifestyle clinic where there is a person they can talk to and who can answer questions.

In contrast to GPs, nurses were more interested, and looked forward to receiving the tool with cautious anticipation; they seemed curious and expressed positive expectations. The overall attitude regarding changes was positive and curious, and they seemed eager to develop their work.

But we know that this is good, just like I said before. This is focus, this health thing ... so it's kind of cool when new things like this pop up.

Nurses also expressed that they were tired of change, and had an obligation to prioritize in times of heavy work load. Their opinions about working with lifestyle issues were expressed in terms like fun and challenging.

Yes, it really is. Prevention is fun.

Nurses described involvement as discussions at staff meetings, but only about where to place the computer. They did not express being involved in the decision; rather they felt that the decision was already made when they received information about the tool.

I guess we discussed it, but no one really asked if ... it just was said that a ... [lifestyle computer] would arrive.

The nurses concluded that the information given at the introduction of the tool was good, but had not reached all individuals.

Among NAs there were expressions of positive expectations, but also a sense of acceptance without enthusiasm. Their opinions about change described openness to innovative changes, but more of just letting things happen combined with reluctance to change when no involvement in the decision was allowed. They talked about addressing lifestyle issues and stressed the importance, but they did not show a commitment to the task and pointed out that professions other than themselves were more appropriate for addressing lifestyle issues.

And then it's also the case that we have this thing with PAR [Physical Activity Referrals] now, and so on, and we have a health coordinator who works with lifestyle issues so ... the doctors refer patients to them.

Discussions about where to place the computer were described by the NAs, but there was no feeling of involvement in the decision; they shared the nurses' opinion that the decision was already made when information was provided. NAs showed little interest in the information about the new tool, but concluded that the quality of the information was good.

### Opinions about the lifestyle test

The opinions about the lifestyle test mentioned by the staff groups involved the compatibility and advantages categories. Managers described the new tool as compatible with PHC, and perceived that it is currently topical to work with lifestyle issues and to use technical solutions to do this.

I think the community is ready for this, absolutely. It's all over the place now ... computers. So, yes, I think so. I think it's positive.

Managers saw several advantages with the computer-based tool; it was perceived as a complement to existing routines, but they were aware that some of the GPs did not consider the computer a valuable tool.

GPs themselves expressed reluctance by pointing out that the computer-based test could be used by other staff groups, such as nurses and NAs.

Nurses' conversations with patients are sometimes a little more informal, at those times when they're there and taking blood pressure and things like that ... when they're lying down and waiting, resting and so on [...] It's kind of a good time to tell them about the lifestyle computer, I think.

In their own work, the GPs saw no use for it; they had other routines, such as referring to a lifestyle clinic at the unit, and feared that the computer-based test could generate extra work. However, a number of advantages were also mentioned by GPs, primarily that patients could perform the test spontaneously and that the test encourages self-care.

You can see that people are using it anyway even without ... that some people do it without anyone having to ask them to. I think that we have a little advantage there, I kind of like it. Even if we forget to mention it, there are still some who walk past it and play with the buttons a bit.

Nurses found the computer compatible with their work and with PHC as a whole but also mentioned advantages for NAs. Some nurses preferred talking directly to their patients about lifestyle and said that patients are often too busy to perform a test. Nurses responsible for lifestyle issues did not seem to want to incorporate the computer-based test into their routines. Nurses, in general, seemed to have embraced the idea of using the computer as a technical aid in their daily work, and also mentioned advantages and possibilities with the computer, for example, advantages for patients completing the test.

I most often ask those who are going to meet me to fill it in and say that we can talk about it after ... But if they don't want to show it to me or talk about it, then it's no problem for me.

Less compatibility was perceived among NAs, as they thought referring patients to the test required further contact with them, and they somehow seemed to lack the confidence to discuss lifestyle issues.

We have been bad at referring, I believe ... and we are not ... no I feel I am not in the right group to refer [patients to the computer].

All groups were concerned that the test only addressed two lifestyle areas, and technical problems were mentioned by all groups except GPs.

### Opinions about usage

Staff opinions about how the new tool had been used involved the following categories: performance, obstacles and solutions.

Informants in all professional groups and managers realized that fewer patients than expected had performed the computer-based test. Managers seemed surprised by this fact, and also expressed feelings of disappointment and resignation. They reflected on difficulties in implementing new routines, and saw the fact that work in PHC is carried out in a very traditional way as hindering work with lifestyle issues.

Our average work day is quite traditional in nature and when you're in a transition phase, looking for new work methods ... because at the same time you're still working according to the old system.

In general, GPs seemed unconcerned about bad performance. They were obviously aware that very few GPs referred patients to the test but expressed no self-criticism; instead they highlighted that other staff groups do refer patients to the computer. Informants in the GP group also seemed indifferent about written feedback (provided by the research team) and posters that were used to encourage referral to the computers.

But we haven't discussed that computer much at all. I don't think anyone has really seen it as being their responsibility.

The main obstacle regarding the use of the new tool mentioned by GPs was forgetting, and it seemed that the reason for forgetting was that they did not find the tool very useful. When GPs discussed possible suggestions for addressing the low usage of the computer, they seemed more anxious to improve statistics than provide a preventive service to patients.

Nurses, on the other hand, expressed a high level of self-criticism; they had an ambition to refer patients, and were disappointed that they rarely did.

I've been really bad at using it. I try to remember, but I have an awful lot of other things to discuss, too ....

The lack of established routines was also seen as an important obstacle.

But you have to find the right forum for it ... I know we've reminded people when we've had low referral rates. We've mentioned it a couple of times at staff meetings and reminded people not to forget the lifestyle computer ... So, that we've done.

Nurses presented several constructive suggestions for improvement, all in order to make the preventive service provided by the test available to more patients. They also noted, with satisfaction, that NAs, at least at some units, referred patients more frequently.

I know ... at this unit it's the nurse assistants, isn't it [who are best at referring patients to the computer].

The informants in the nurse group were concerned that the written feedback (provided by the research team) did not reach all staff members because of structural issues at the unit.

NAs were aware of the low performance in their own group, but referring patients to the computer did not seem to be prioritized, and forgetting and lack of time were mentioned as obstacles. They also asked for livelier discussions in the staff group, and were disappointed that managers did not stimulate this.

But then there's also the fact that we don't ever have any time. There's no special time set aside for these kinds of things and so it ends up taking a back seat, and we're supposed to be able to find time to fit this in somewhere, too.

Suggestions for improvements made by NAs included displaying signs and advertisements about the computer-based test in order to make referral unnecessary, thus expressing reluctance to incorporate the computer as a working tool.

I just thought about that now, that perhaps we could do that [set up a sign encouraging patients to test themselves] ... so we wouldn't have to ... so that they themselves get the thought in their minds that they can go to [the computer].

NAs at one of the six units did show enthusiasm and seemed to have embraced the idea of using the computer-based lifestyle tool as it was intended.

## Discussion

The purpose of this study was to explore how managers and professionals experienced the implementation of a new working tool in PHC. The main finding was that managers were visionary, GPs reluctant, nurses open and NAs indifferent with regard to the implementation. Existing values, beliefs and behaviour within the professional subcultures seemed to influence the perceptions of the implementation.

Health care organizations are known to be heterogeneous, with a number of different professions working together, side by side or in teams. Each staff category tends to create their own subculture, and a hierarchical structure is present among the professions, with physicians being the most powerful subculture [4-8]. The creation of subcultures can be explained using social influence theories, which claim that it is not a conscious consideration of advantages that predicts a specific behaviour, but routines observed in others and the social norms of the network [24,25].

Medical faculties, in contrast to the more behavioural approach at departments of nursing, have traditionally based their education on biological research, thus representing different paradigms in health care and resulting in different values among professionals [26,27]. Thus, there are differences in professional education and social influences within each profession.

Some medical specialties have been found to be perceived as more prestigious than others, with preventive medicine a less prestigious area [28]. If a new field of activity is perceived as burdensome or of negative prestige, the professions try to get rid of it [5]. Probably that could explain why participants in our study found the tool for lifestyle intervention more suitable as a working tool for other professionals. GPs were passing it over to nurses, NAs or physical therapists; nurses appreciated the tool, but also mentioned the possibilities it has for NAs; NAs suggested that computers should preferably be available for patients to perform self-initiated tests. It has also been suggested that the implementation of screening and intervention for unhealthy behaviours is hindered by clinicians' unwillingness to allow increased involvement by nurses or medical assistants in care [29]. Our study points in the opposite direction, indicating that physicians do invite other professional groups to work on the subject. One explanation to this could be that the subject is not perceived to be prestigious among the GPs.

When new ways of working are imposed, well-established groups sometimes show a kind of protectionism as a defence against the perceived threat, causing resistance to change [30]. GPs in former research have expressed certain ambiguity regarding change [31]. In our study, changes seemed to be perceived as threatening among GPs; with regard to the new tool, they seemed to fear the possibility of an increased work load as a result of patients performing the test. It is known that GPs are struggling to meet increasing patient and administrative demands, and probably it is natural that they fear additive tasks [11]. An increased work load, however, is a common problem among all professionals in PHC. In the development of the computer-based concept, the intention was to provide a self-administrated screening and intervention tool in order to decrease work load and facilitate a focus on lifestyle issues.

Preventive issues have traditionally been central to nursing, and with modern technology, the options increase [32]. Nurses in our study believed in the new tool; they saw possibilities and thought it would be fun and exciting to receive it. This could be one explanation why the new tool was perceived compatible by nurses. However, regarding behaviour, not even the nurses used the tool as intended, probably due to structural implementation factors, discussed further below.

NAs have the lowest level of education among health professionals in Swedish PHC, and in many cases they are the smallest group. The work of NAs is strongly patient-oriented, but also supports other staff groups, and they are not expected to take initiatives [33]. In the present study, NAs expressed remoteness from changes experienced at the unit. They did not feel involved in the decision; they act as observers, not very engaged in what is going on, and most of them did not express any willingness to incorporate the new tool in their daily work.

The manager's role is challenging, and managers' attitudes have been shown to be crucial for organizational change [1]. We found that managers were open and optimistic; they had great expectations and were disappointed in the results. As an explanation for the disappointing results, managers mentioned the importance of a special person in charge at the unit, something that had obviously failed at the local level. It is known that a key opinion leader has the potential to play an important role in changing clinical practice [1]. Managers also called for a higher degree of obligation to refer patients to the lifestyle test. Incentives or reinforcement, which could be either positive or negative, are described by Nutley et al. as a key strategy to improve the use of research [34].

When the new working tool was introduced, the information given to the staff seems to have been provided in a suboptimal way; the different professional groups and their needs were not taken into account. Providing information in groups according to profession, as also suggested by the managers, may have increased curiosity about the new tool and influenced usage in a positive way. Tailored information and discussion in professional groups could also have increased the perception of involvement, which is an important factor that influences behaviour [8]. Only managers had the feeling that staff were involved in the decision process; the professional groups had another opinion. This shows that discussion at staff meetings is not enough to provide a perception of involvement.

One explanation for the varying experiences of the implementation between the groups could be differences in gender. In the GP group, almost half of the participants were men, while all other participants except one nurse were women. It has been reported previously that men are less positive about health promotion in health services than women [35].

### Methodological considerations

The six PHC units participating in the study varied in size, catchment area and services provided. The differences in contextual factors reflect the situation in Swedish PHC and help to widen the perspective, and thus could be regarded as a strength. In order to expand knowledge about how the different subcultures in health care affect implementation issues, we incorporated all possible professions, even those with only a few staff members, which we thought would add an important dimension to the study, making the picture more complete. However, most of the informants were nurses and GPs, corresponding to the number employed. No physiotherapists chose to participate, which was unfortunate, since physiotherapists in PHC have previously been found to be willing to increase work on health promotion [35]. Overall, too few APs participated to be considered in the analysis.

The time used for the interviews was limited. The staff, out of convenience, participated in interviews during their lunch hour. In the study, the implementation process concerned a specific innovation in the lifestyle intervention area and implementation of other innovations may follow other patterns. Also, the lifestyle tool was perceived by all groups as being too limited.

A qualitative method, as used in this study, aims to explore participants' opinions and experiences regarding a predetermined subject. The findings serve to expand knowledge about how the subject is perceived, knowledge that may be transferable to other circumstances [36]. The authors in this article have different experiences and pre-understandings, which was perceived as valuable in the analytic process.

## Conclusions

This study indicates that values, beliefs and behaviour associated with the existing subcultures in PHC influence how the implementation of an innovation is perceived by managers and the different professionals. Managers put a high value on addressing lifestyle in PHC; they believed that the new tool could facilitate this work and welcomed it at their units, had high expectations and showed a positive and visionary attitude to the implementation. GPs also valued the task of addressing lifestyle issues, but their independence was more important, which was also shown in a negative attitude to change. They believed in their existing models of fulfilling the task, and saw no need for the tool. The implementation of the tool was perceived as threatening, and GPs behaviour was characterized by reluctance. Nurses showed an open attitude to change; they valued lifestyle issues and they believed that the tool could be of benefit. However, they also put high value on existing routines. They were open to the implementation, but failed to live up to their own expectations. NAs did value lifestyle issues, but in general did not see their own contribution to the task. They did not feel confident with it, and believed it was the responsibility of other professionals. The implementation of the new tool was met with indifference. In PHC, an organization with several subcultures and an established hierarchical structure, an implementation strategy aimed at all professional groups did not seem to result in a successful uptake of the new method. A suggestion for further research could be to consider the different subcultures and tailor the implementation activities to meet professional needs, as was suggested by the informants in this study.

## Competing interests

The authors declare that they have no competing interests.

## Authors' contributions

SC participated in the design of the study, performed the interviews, was responsible for the data analysis and wrote the first draft of the manuscript. AA participated in the design of the study, took part in analysing the data and in preparing the manuscript. ML participated in the design of the study, took part in analysing the data and in preparing the manuscript. All authors read and approved the final manuscript.

## Authors' information

S.C. is a Registered Physical Therapist, MPH, currently a PhD Student at the Department of Medical and Health Sciences, Division of Community Medicine, Linköping University, SE-581 83 Linköping, Sweden. A.A. is a Health Economist, PhD, working as a research supervisor at the R&D Department of Local Health Care in Östergötland, Linköping University, SE-582 24 Linköping, Sweden. M.L. is a Registered Nurse, PhD, working as a research supervisor at the R&D Department of Local Health Care in Östergötland, Linköping University, SE-582 24 Linköping, Sweden.

## Pre-publication history

The pre-publication history for this paper can be accessed here:

http://www.biomedcentral.com/1472-6963/11/195/prepub
